# Supplementary material for: Causal Relationship between Aging and Anorexia Nervosa: A White-Matter-Microstructure-Mediated Mendelian Randomization Analysis
Source: Biomedicines. 2024 Aug 16;12(8):1874. doi: 10.3390/biomedicines12081874 (PMC11351342; doi:10.3390/biomedicines12081874)
Supplement: Supplementary file 1 [file biomedicines-12-01874-s001.zip › Supplementary Figure/Figure S8/Leaveoneout plot of MR analysis for TL to ubm-b-1646.pdf]

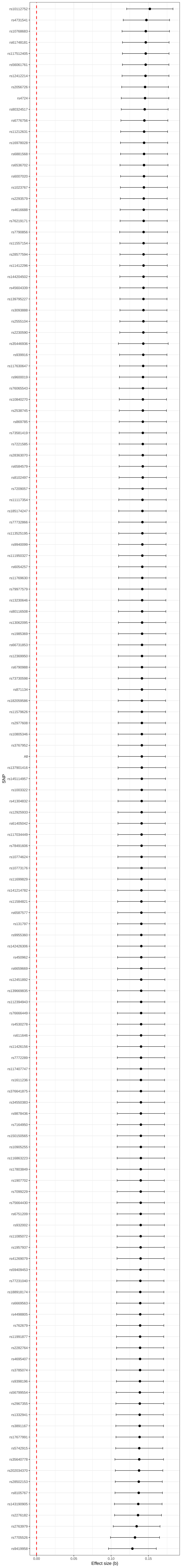

rs10112752
rs4731541
rs10768683
rs61748181
rs117512405
rs56061761
rs12412214
rs2056726
rs4724
rs80324517
rs6776756
rs11212631
rs16978028
rs6881568
rs6536702
rs6007020
rs1023767
rs2293579
rs4616688
rs76219171
rs7790856
rs11557154
rs28577594
rs11422296
rs144204502
rs45604339
rs139795227
rs3093888
rs2555104
rs2230590
rs35446936
rs939916
rs117630647
rs9600019
rs76065543
rs10840270
rs2538745
rs869785
rs73581419
rs7221585
rs28363070
rs6584579
rs8102497
rs7209057
rs11117354
rs185174247
rs77732866
rs113525195
rs9940099
rs111950327
rs6054257
rs11769630
rs79977579
rs13230646
rs80116508
rs13062095
rs1985369
rs66731853
rs12369950
rs6790988
rs73730598
rs871134
rs182059586
rs11579626
rs2977608
rs10805346
rs3767952
A8
rs137901416
rs145114957
rs1003322
rs41304832
rs12925933
rs61405042
rs117034449
rs78491606
rs10774624
rs10773176
rs11699829
rs141214782
rs11584821
rs6587577
rs131797
rs9955360
rs142426306
rs450962
rs6659669
rs12451892
rs139669835
rs112394943
rs76666449
rs4530278
rs611646
rs11426156
rs7772289
rs117407747
rs1611236
rs376641875
rs34550383
rs9878436
rs7164950
rs150150565
rs10905255
rs116863223
rs17803849
rs1907702
rs7099229
rs75664430
rs6751209
rs932002
rs11085072
rs1957937
rs41269079
rs59409453
rs77231040
rs188918174
rs6669563
rs4498805
rs762679
rs11991877
rs2282764
rs4695407
rs3785074
rs9398196
rs56799554
rs2967355
rs1332941
rs3891167
rs17677991
rs5742915
rs35640778
rs202034370
rs28502153
rs8105767
rs143190905
rs2276182
rs2763979
rs7705526
rs9419958
SNP
0.00
0.05
0.10
0.15
Effect size (b)
